# Role of ultrasonography in early diagnosis of congenital extrahepatic portosystemic shunt

**DOI:** 10.1259/bjrcr.20150266

**Published:** 2016-05-25

**Authors:** Kithir Mohamed Anwer Sadat

**Affiliations:** Department of Radiology, Tiruvarur Medical Centre, Tiruvarur, India

## Abstract

A 1-year-old male presented to our unit for ultrasonography of the abdomen with a history of fever and diarrhoea for 2 days. The clinical examination was normal. Ultrasound of the abdomen showed a small portal vein and splenomesenteric venous shunt into the left renal vein that was consistent with a Type II congenital extrahepatic portosystemic shunt (CEPS). Doppler sonography confirmed the flow towards the left renal vein. No abnormal findings were observed in the liver and rest of the abdominal organs. CEPS is an uncommon anomaly in which the portomesenteric blood drains into a systemic vein, bypassing the liver through a complete or partial shunt. It is usually diagnosed incidentally during investigation for other causes. Morgan and Superina (Congenital absence of the portal vein: two cases and a proposed classification system for portasystemic vascular anomalies. *J Pediatr Surg* 1994; **29**: 1239–41) proposed a classification for these shunts in 1994 that divided these anomalies into two types. In Type I CEPS, the intrahepatic portal venous supply is absent, whereas in Type II, the intrahepatic portal venous supply is preserved. This case highlights the role of ultrasonography in the early diagnosis of CEPS.

## Case presentation

A 1-year-old male presented to our unit for ultrasonography of the abdomen with complaints of fever and diarrhoea for 2 days to rule out mesenteric adenitis. The clinical examination was normal, with no signs of dehydration. There was no significant clinical history.

## Investigation

**Figure 1.**
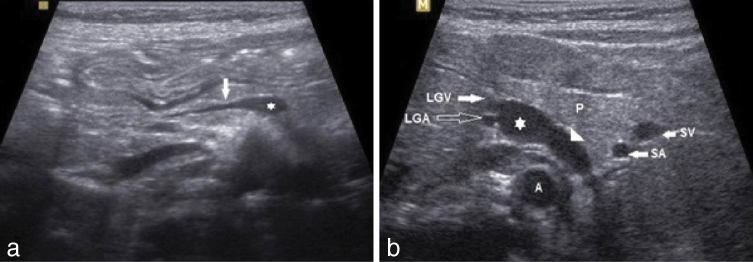
(a) An oblique sonogram of the upper abdomen shows the hypoplastic portal vein (arrow) arising from the splenomesenteric confluence (asterisk). (b) The transverse sonogram shows the shunt vessel (arrowhead) from the splenomesenteric confluence (asterisk) extending posterior to the pancreas and lying along the left side of the aorta. A, aorta; LGA, left gastric artery; LGV, left gastric vein; P, pancreas; SA, splenic artery; SV, splenic vein.

**Figure 2.**
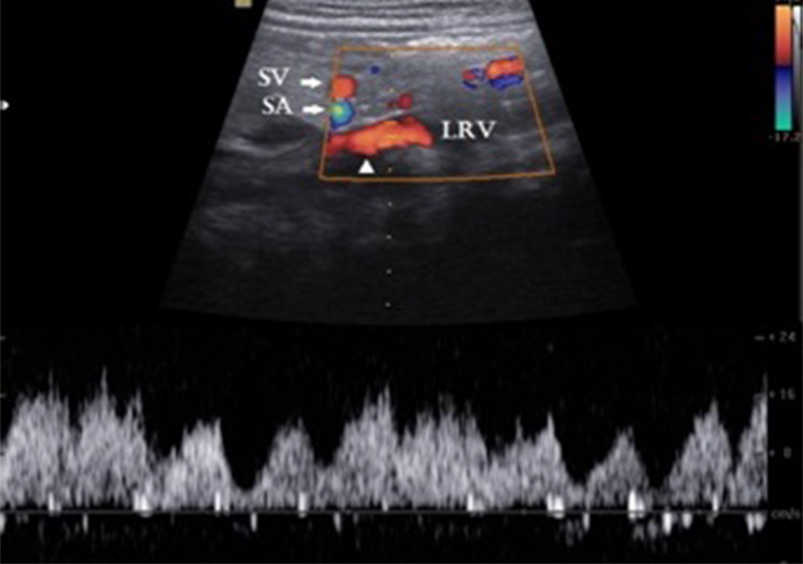
Duplex Doppler sonography in longitudinal axis shows the shunt vessel (arrowhead) with pulsatile flow owing to the transmitted cardiac periodicity. Flow direction is towards the left renal vein. LRV, left renal vein,; SA, splenic artery; SV, splenic vein.

On ultrasonography, the bowel was normal and there was no mesenteric adenitis. Incidentally, he was found to have a small-sized portal vein arising from the side of the splenomesenteric venous confluence (Figure 1a). There was a vessel noted along the left side of the aorta (Figure 1b). This vessel was seen as a continuation of the splenomesenteric venous confluence and had a curved course, extending posterior to the pancreas, splenic vessels and ending in the left renal vein. Duplex Doppler sonography showed venous flow towards the left renal vein (Figure 2). The flow in the small portal vein was low and in the hepatopetal direction.The liver appeared normal in size and texture and there were no secondary signs of portal hypertension such as splenomegaly or ascites. Based on the features described above, a diagnosis of congenital extrahepatic portosystemic shunt (CEPS) Type II was made.

**Figure 3.**
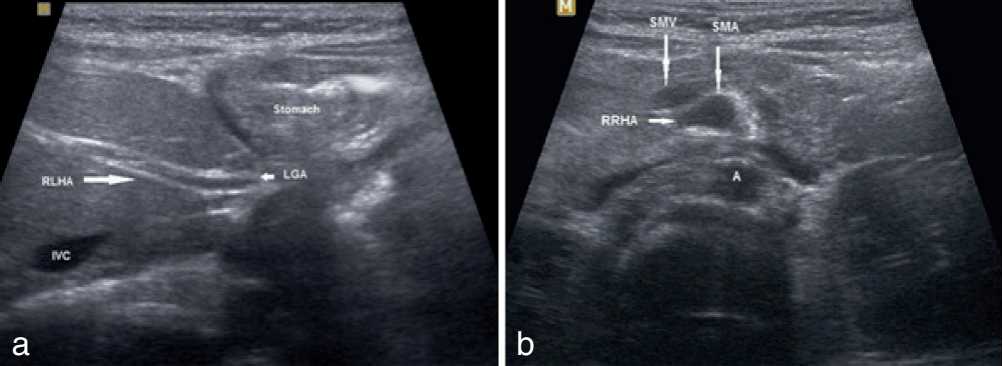
(a) The left hepatic artery (long arrow) arises from the LGA and runs through the fissure of the ligamentum venosum and is termed the RLHA. (b) The right hepatic artery (short arrow) arises from the SMA and is termed the RRHA. A, aorta; IVC, inferior vena cava; LGA, left gastric artery; RLHA, replaced left hepatic artery; RRHA, replaced right hepatic artery; SMA, superior mesenteric artery; SMV superior mesenteric vein.

**Figure 4.**
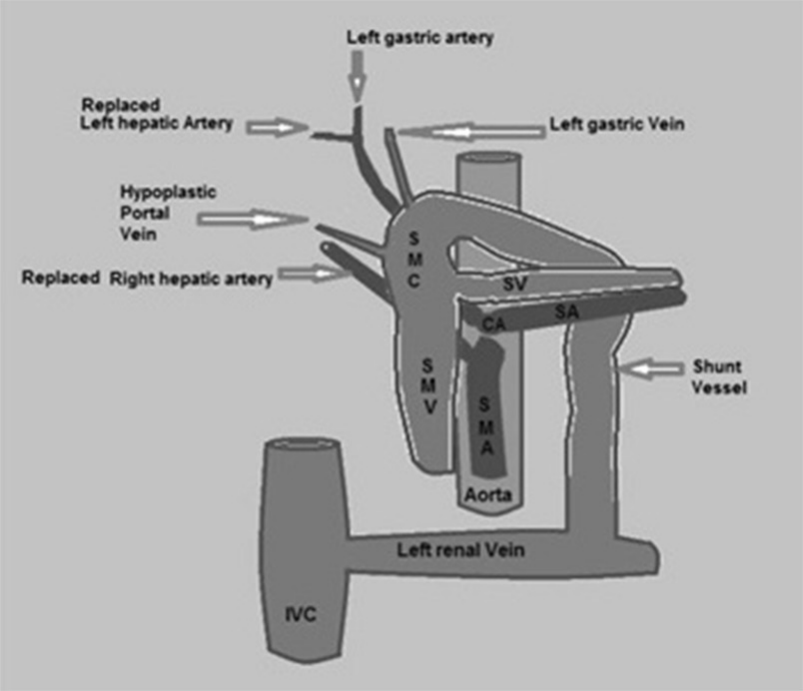
Schematic illustration of the anomaly shows the hypoplastic portal vein, shunt vessel connecting the splenomesenteric confluence with the left renal vein (Type II extrahepatic portosystemic shunt), the left hepatic artery originating from the left gastric artery (replaced left hepatic artery) and the right hepatic artery originating from the superior mesenteric artery (replaced right hepatic artery). CA, celiac axis; SA, splenic artery; SMA, superior mesenteric artery; SMC, splenomesentric confluence; SMV, superior mesenteric vein; SV, splenic vein.

Furthermore, the liver showed variant arterial supply. The left hepatic artery originated from the left gastric artery (Figure 3a) and the right hepatic artery from the superior mesenteric artery (Figure 3b). Schematic illustration of the findings are shown in Figure 4.

## Differential diagnosis

Acquired causes of portosystemic shunt such as cirrhosis of the liver or portal vein thrombosis need to be excluded from the differential diagnosis. CEPS cases typically do not show secondary features of portal hypertension such as splenomegaly or ascites. In our ultrasonographic examination, there was no splenomegaly or ascites and the liver was normal. The small-sized portal vein showed no signs of luminal thrombus.

## Treatment

The case was discussed by a board of doctors, including a paediatric surgeon, and an abdominal MRI and MR angiography were suggested. Unfortunately, the parents declined to let the patient undergo an MRI and therefore further evaluation was impossible. Counselling was given to the parents about the anomaly and management plan. Periodic clinical follow-up and ultrasonography was advised.

## Discussion

CEPS is an unusual anomaly in which the portomesenteric blood drains into a systemic vein, bypassing the liver through a complete or partial shunt. CEPS was reported in 1793 by Abernethy.^1^ In 1994, Morgan and Superina^2^ classified CEPS into two types based on the presence or absence of intrahepatic portal venous supply. In Type I CEPS, there is an absence of intrahepatic portal branches, with complete diversion of the portal blood into the systemic circulation. Type I CEPS shunts are further classified into Type Ia and Ib. In Type Ia CEPS, the splenic vein (SV) and superior mesenteric vein (SMV) drain separately into a systemic vein, whereas in Type 1b, the SV and SMV drain together into a systemic vein after joining to form a common trunk. In Type II CEPS, the intrahepatic portal vein is intact but some of the portal flow is diverted into a systemic vein through a shunt.^2^ The most common draining systemic vein is the inferior vena cava (IVC); the others are the renal, iliac and azygos vein or the right atrium.^3^

The portal vein develops from the infrahepatic part of the right and left vitelline veins. IVC has a complex embryologic development from the cardinal systems of veins. In the early embryonic stage, the subcardinal and vitelline veins have anastomotic channels between them. The persistence of these interconnections or the right vitelline vein has been proposed to cause CEPS.^4^

Type I CEPS usually occurs in females. The Type I patients are typically young at presentation because of their frequent associations with anomalies such as congenital heart disease, situs ambiguous, polysplenia, skeletal anomalies and genitourinary system malformations. Type II CEPS has no gender preference. As there are less frequently associated anomalies in Type II CEPS, presentation is usually later in life.^4^

Most children with CEPS are usually asymptomatic. Liver dysfunction and hepatic encephalopathy are the common complications of CEPS that usually develop during adulthood. However, the child can develop encephalopathy following a precipitating event such as gastrointestinal bleeding, constipation or systemic infection. CEPS is usually diagnosed incidentally during investigation of non-specific liver disease or evaluation of other anomalies and encephalopathy. It can also be detected by a prenatal ultrasound and following evaluation of positive galactosaemia screening test. The clinical manifestation can occur owing to systemic shunting of the splanchnic blood that results in abnormal development, function and regeneration of the liver. Elevated serum metabolites such as ammonia can result in encephalopathy. CEPS cases are often associated with benign liver nodules and regenerative nodular hyperplasia but hepatoblastoma and hepatic cellular carcinoma have also been reported in CEPS.^4^

As ultrasonography is usually the initial imaging investigation for non-specific liver disease and other abdominal complaints, familiarity with the imaging features of CEPS may help in making an early diagnosis. Ultrasound with colour Doppler can demonstrate presence of the shunt, the type and the direction of flow. Once the anomaly has been identified with ultrasound, a CT scan or MRI should be performed to confirm the diagnosis. CT/MR angiography could provide additional information about the hepatic vascular abnormalities. In order to avoid ionizing radiation to paediatric patients, MRI or MR angiography is preferable, but CT angiography is considered to be superior in detecting the fine vascular details because of the good spatial resolution. Conventional angiography is not usually required for the diagnosis of CEPS but prior to starting definitive treatment, it is necessary to define the shunt anatomically and functionally. Iodine 123 iodoamphetamine transrectal portal scintigraphy is useful in calculating portosystemic shunt ratio in Type II CEPS but is not routinely advocated. Shunt ratio is useful in identifying patients at risk of developing encephalopathy. A shunt ratio of more than 5% is abnormal. Patients with a shunt ratio of 30–40% are prone to developing encephalopathy following the precipitating events and those with more than 60% shunt ratio are at high risk of developing spontaneous encephalopathy.^4^

As the children with CEPS are usually asymptomatic, watchful waiting is indicated. Definitive treatment is recommended when the patient develops symptoms. Careful clinical and ultrasound follow-up should be performed until definitive treatment. Therapeutic options depend on the type of shunt. Preoperatively, it is necessary to evaluate the complete anatomical details of the shunt by catheter angiography. The extremely hypoplastic portal veins distal to the shunt are better visualized by catheter angiography than conventional CT angiography; catheter angiography can distinguish a true Type I shunt having absolute lack of opacification of intrahepatic portal branches from a Type II shunt with very small portal vein branches.^5^

Liver transplantation is the therapeutic option in Type 1 CEPS, when the patient develops encephalopathy.^6^ Kanazawa et al^7^ proposed a new classification based on severity of hypoplasia of the intrahepatic portal venous system identified by angiography and stressed the necessity to re-evaluate the need for liver transplantation. Larger studies are required to validate these results. In Type II CEPS, surgical or endovascular shunt occlusion in a single or staged approach can be an effective treatment. The objective is to close the shunt, while avoiding an increase in portal pressure. Temporary closing of the shunt by balloon occlusion and observing the portal venous pressure before and after the occlusion is a useful test to differentiate between patients who can tolerate immediate shunt ligation and those who could develop severe portal hypertension and would need a staged approach.^5,8^

Here we have reported a Type II CEPS coexisting with the left hepatic artery originating from the left gastric artery and the right hepatic artery originating from the superior mesenteric artery. Although the variant hepatic arterial system is a common anomaly, its coexistence with CEPS becomes significant when liver transplantation or surgical shunt occlusion is indicated.

Ultrasonography is a useful tool to identify and classify CEPS, detect associated anomalies and during follow-up; the assessment of flow direction via Doppler is also an advantage.

## Learning points

1. Familiarity with the imaging features of CEPS is important, as most of the cases are detected incidentally by ultrasonography.
2. In Type I CEPS, the portal blood is completely directed into the systemic circulation as the hepatic portal venous supply is absent. In Type II CEPS, a part of the portal blood is diverted into the systemic circulation. The Type I patients are typically young at presentation, whereas the Type II CEPS patients usually present later.
3. CEPS should be differentiated from acquired causes of portosystemic shunt such as liver disease and portal vein thrombosis.
4. Features of portal hypertension such as splenomegaly or ascites are typically not seen in CEPS.
